# Role of gadolinium enhanced cardiac magnetic resonance in recent onset non-ischemic cardiomyopathy: a systematic review and metanalysis

**DOI:** 10.1186/1532-429X-17-S1-P173

**Published:** 2015-02-03

**Authors:** Diego A Eifer, Felipe S Torres, Murilo Foppa

**Affiliations:** Radiology, Hospital de Clinicas de Porto Alegre, Porto Alegre, Brazil; Internal Medicine: Cardiology, Hospital de Clinicas de Porto Alegre, Porto Alegre, Brazil

## Background

Cardiac magnetic resonance (CMR) late gadolinium enhancement (LGE) and early gadolinium enhancement (EGE) are powerful diagnostic tools for acute cardiomyopathies. LGE is associated with impaired prognosis in ischemic and in chronic non-ischemic cardiomyopathy (NICM), but the impact of gadolinium enhanced CMR on prognosis in recent onset NICM is less well established. We performed a systematic review with metanalysis to investigate the prognostic role of an abnormal contrast enhanced CMR in patients presenting with recent onset NICM.

## Methods

From January 1994 to July 2014, Pubmed, EMBASE and additional bibliographic sources were searched for publications using contrast enhanced CMR (EGE and LGE) in patients with recent onset NICM, defined as symptoms onset within 6 months of presentation, including myocarditis, acute chest pain syndromes with normal coronaries, and idiopathic acute cardiomyopathy. We included articles that assessed total mortality and/or composite cardiovascular events (mortality plus any combination of cardioverter defibrillator implantation, cardiac transplantation, hospitalization or worsening heart failure) with a follow-up of least 3 months after the initial presentation. Data were independently extracted by 2 authors and pooled risk ratios (RR) were calculated using the DerSimonian and Laird random-effects method.

## Results

**Figure 1:**
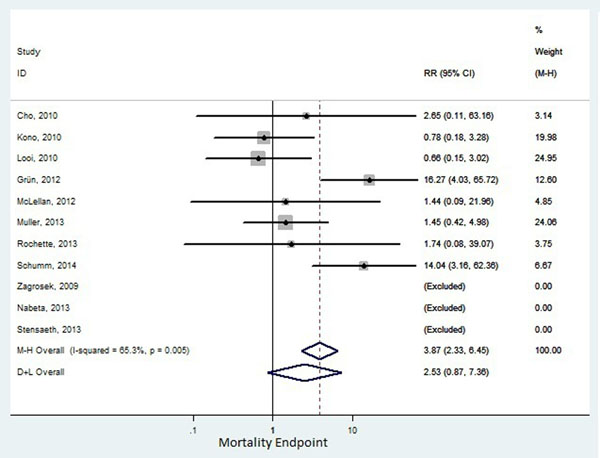
Mortality endpoint

**Figure 2:**
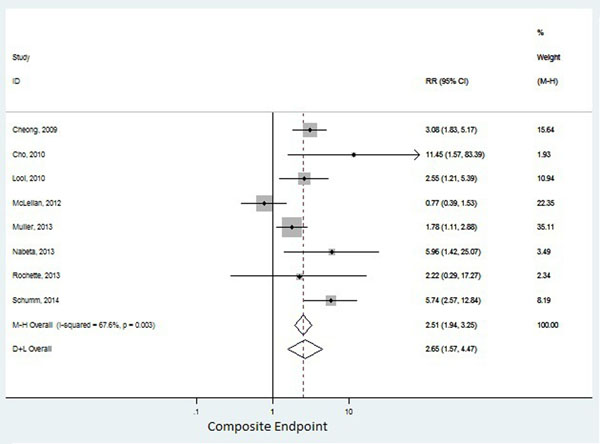
Composite endpoint

Eleven publications (1249 patients) were identified for the mortality endpoint and 8 (1151 patients) for the composite endpoint, with an average follow-up varying from 3 to 56 months. Five studies had myocarditis as the primary diagnostic hypothesis and seven had non-ischemic cardiomyopathy. Prevalence of abnormal CMR varied from 28 to 64%, which was defined by LGE in 9 studies and by EGE or a combination of both in the remaining studies. Abnormal CMR had a pooled RR of 2.53 (95%CI: 0.87-7.4) for total mortality (figure 1), and a pooled RR of 2.65 (95%CI: 1.57-4.47) for composite endpoints (figure 2), with significant heterogeneity, I2=65.5%;P=0.005, and I2=67.6%;P=0.003, respectively.

## Conclusions

In patients presenting with recent onset NICM, the presence of an abnormal contrasted enhanced CMR is associated with a statistically significant increased risk ratio for composite endpoints. These findings may help identify those patients with a worse prognosis and increased use of medical resources.

## Funding

N/A.

